# *In Vitro* Evaluation of Biphasic Calcium Phosphate Scaffolds Derived from Cuttlefish Bone Coated with Poly(ester urea) for Bone Tissue Regeneration

**DOI:** 10.3390/polym15102256

**Published:** 2023-05-10

**Authors:** Patrícia Pereira, Ana S. Neto, Ana S. Rodrigues, Inês Barros, Catarina Miranda, João Ramalho-Santos, Luís Pereira de Almeida, José M. F. Ferreira, Jorge F. J. Coelho, Ana C. Fonseca

**Affiliations:** 1 IPN, Instituto Pedro Nunes, Associação para a Inovação e Desenvolvimento em Ciência e Tecnologia, Rua Pedro Nunes, 3030-199 Coimbra, Portugal; 2 CEMMPRE, Department of Chemical Engineering, University of Coimbra, Rua Sílvio Lima-Pólo II, 3030-790 Coimbra, Portugal; 3 Department of Materials and Ceramic Engineering/CICECO–Aveiro Institute of Materials, University of Aveiro, 3810-193 Aveiro, Portugaljmf@ua.pt (J.M.F.F.); 4 CNC-Center for Neuroscience and Cell Biology, University of Coimbra, 3004-504 Coimbra, Portugal; 5 CIBB-Center for Innovative Biomedicine and Biotechnology, University of Coimbra, 3004-504 Coimbra, Portugal; 6 III-Institute for Interdisciplinary Research, University of Coimbra, 3030-789 Coimbra, Portugal; 7 DCV-Department of Life Sciences, University of Coimbra, 3000-456 Coimbra, Portugal; 8 Faculty of Pharmacy, University of Coimbra, 3000-548 Coimbra, Portugal; 9 Viravector-Viral Vector for Gene Transfer Core Facility, University of Coimbra, 3004-504 Coimbra, Portugal

**Keywords:** cuttlefish bone, biphasic calcium phosphate, polymer coatings, *in vitro* cell culture, osteogenic differentiation

## Abstract

This study investigates the osteogenic differentiation of umbilical-cord-derived human mesenchymal stromal cells (hUC-MSCs) on biphasic calcium phosphate (BCP) scaffolds derived from cuttlefish bone doped with metal ions and coated with polymers. First, the *in vitro* cytocompatibility of the undoped and ion-doped (Sr^2+^, Mg^2+^ and/or Zn^2+^) BCP scaffolds was evaluated for 72 h using Live/Dead staining and viability assays. From these tests, the most promising composition was found to be the BCP scaffold doped with strontium (Sr^2+^), magnesium (Mg^2+^) and zinc (Zn^2+^) (BCP-6Sr2Mg2Zn). Then, samples from the BCP-6Sr2Mg2Zn were coated with poly(ԑ-caprolactone) (PCL) or poly(ester urea) (PEU). The results showed that hUC-MSCs can differentiate into osteoblasts, and hUC-MSCs seeded on the PEU-coated scaffolds proliferated well, adhered to the scaffold surfaces, and enhanced their differentiation capabilities without negative effects on cell proliferation under *in vitro* conditions. Overall, these results suggest that PEU-coated scaffolds are an alternative to PCL for use in bone regeneration, providing a suitable environment to maximally induce osteogenesis.

## 1. Introduction

Bone defects and bone destruction caused by disease (osteoporosis, bacterial infections, osteoarthritis and tumor) or accidental factors (car accidents and trauma) are increasingly common and have a huge impact on a patient’s quality of life [1]. Because of the limited availability of biological bone substitutes, several tissue engineering strategies have been widely considered in the reconstruction of vascularized bone tissue and in the treatment of bone defects, namely, the combination of cells, biological molecules and/or (bio)materials [2]. Regarding the materials, they must be biocompatible and provide a suitable environment to accommodate a sufficient number of cells at the injury site and, therefore, promote cell adhesion, proliferation and differentiation without adverse effects on the host tissue [3]. It should preferably be biodegradable at a similar rate to that of bone formation, and the resulting by-products should be non-toxic (biosafety) [4,5].

Among the various biomaterials used as bone graft substitutes, calcium phosphate (CaP)-based biomaterials have received special attention in recent years due to their chemical similarity to the inorganic matrix of natural bone tissue [6]. CaP-based biomaterials represent a large family of materials formed by the reaction of calcium and phosphate ions. The most common CaP-based biomaterials are hydroxyapatite [HAp, Ca_5_(PO_4_)_3_OH)], β-tricalcium phosphate [β-TCP, Ca_3_(PO_4_)_2_] and their mixtures, which are called biphasic calcium phosphates (BCPs) [7]. HAp offers biocompatibility, chemical stability in body fluids, and microporosity but is poorly resorbable and fragile, whereas β-TCP is biodegradable and allows a faster release of constituent ions [2,8,9]. Compared with HAp or β-TCP, BCP-based biomaterials have better biocompatibility, superior mechanical properties, higher biological activity, tunable degradation and resorbable properties that provide a physiological environment to support nutrient transport and bone tissue ingrowth [10,11,12,13,14].

It is also known that co-doped CaP-based biomaterials can mimic the chemical composition, functionality and properties of natural bone and can therefore clearly favor the process of osteogenesis and rapid healing [15,16]. The ideal doping content depends on the type of ion used. Several reports can be found in the literature: (strontium (Sr^2+^) [17,18], magnesium (Mg^2+^) [13,19,20], cobalt (Co^2+^) [21,22], copper (Cu^2+^) [22,23], chromium (Cr^3+^) [22], lithium (Li^+^) [24], silicon (Si^4+^) [25,26], cerium (Ce^3+^) [25], nickel (Ni^2+^) [27] and selenium (Se^4+^) [28]). Each has different amounts of doped ions. Sr^2+^ and Zn^2+^ ions are known to promote bone formation by enhancing the osteoclastic resorption process and increasing pre-osteoblastic cell proliferation. Mg^2+^ ions play a role in the activity of osteoblasts and stimulate bone growth [15]. When the amount of doped metal ions is adequate, their release triggers osteogenesis and angiogenesis and enables effective regeneration of bone tissue [16]. On the other hand, excessive accumulation of dopant ions may trigger cytotoxicity and the inhibition of biological activity [29]. Additionally, few studies have investigated the effect of co-doping and its biological relevance; for example, Sr^2+^/Fe^3+^ co-doped BCPs [30], Sr^2+^/Mg^2+^ co-doped BCPs [31] and Ce^3+^/Si^4+^ co-doped BCPs [32]. Basu and et al. showed that BCPs co-doped with Sr^2+^/Fe^3+^ support osteoblast proliferation, while the single-doped BCP (at a dopant content of 10 mol% or higher) exhibited a significant reduction in cell viability [30]. Similar results have been reported for Sr^2+^/Mg^2+^ co-doped BCPs [31] in osteoblasts. Priyadarshini and Vijayalakshmi also proved that dual-doped HAp (Ce^3+^/Si-HAP@5% in a concentration up to 800 µg/mL) has excellent bone-like apatite layer formation on its surfaces and good metabolic activity on the MG-63 692 osteoblast-like cell [32].

Ferreira’s research group was the first to produce HAp from cuttlefish bone, which has a unique porous structure and the ability of the aragonite mineral to be hydrothermally converted to CaPs [11,33]. However, HAp scaffolds derived from cuttlefish bone have poor mechanical properties, especially high brittleness and poor load-bearing capacity. To overcome these low mechanical properties, the scaffolds can be coated with polymers such as hyaluronic acid/gelatin [34], poly(ε-caprolactone) (PCL)/poly(lactide-*co*-glycolide) [35] and PCL [36,37] to achieve the desired functional and physicochemical properties of CaPs. In our recent publications [38,39,40], we reported the preparation and physicochemical characterization of multifunctional BCP scaffolds coated with PCL or poly(ester urea) (PEU). The results showed a significant improvement of the compressive strength of the scaffold, especially with the PEU coating [39]. The conducted studies also showed that the scaffold coated with PEU exhibited an excellent cytocompatibility with hUC-MSCs, promoting their adhesion and proliferation on its surface [38].

Considering the excellent previous results, in this work, we investigate the effect of doping polymer-coated BCP scaffolds with three ions, namely, Sr^2+^, Mg^2+^ and Zn^2+^ (PCL-coated BCP-6Sr2Mg2Zn and PEU-coated BCP-6Sr2Mg2Zn) to promote the differentiation of human mesenchymal stromal cells from umbilical cord matrix (hUC-MSCs) into an osteogenic lineage. Although the effects of these ions on osteoblast functionality are known, their combined effect has never been investigated. To identify the osteogenic responses of the doped scaffolds, the behavior of hUC-MSCs with coated doped scaffolds was examined using the MTS assay, alkaline phosphatase (ALP) staining and Alizarin Red S (ARS) staining.

## 2. Materials and Methods

### 2.1. Preparation of BCP Scaffolds Derived from Cuttlefish Bone

The scaffolds used in this work were prepared and characterized as described elsewhere [25,26,27]. Briefly, cuttlefish bones were carefully cut into small square-shaped pieces (~10 mm × 10 mm × 2 mm) and then subjected to hydrothermal transformation into BCP scaffolds [40]. Undoped (BCP) and doped compositions containing strontium (BCP-6Sr), strontium and magnesium (BCP-6Sr2Mg), strontium and zinc (BCP-6Sr2Zn), or strontium, magnesium, and zinc (BCP-6Sr2Mg2Zn) were prepared. Briefly, di-ammonium hydrogen phosphate (NH_4_)_2_HPO_4_ was used as a starting chemical precursor for phosphorous, while strontium nitrate (Sr(NO_3_)_2_), magnesium nitrate hexahydrate (Mg(NO_3_)_2_.6H_2_O) and zinc nitrate hexahydrate (Zn(NO_3_)_2_.6H_2_O) were used as the starting precursors for strontium, magnesium and zinc. Cuttlefish bones (CBs) obtained from *Sepia officinalis* were used as a source of calcium. The CB pieces with a known amount of CaCO_3_ were mixed with the required amount and concentration of the phosphorous precursor solution to obtain the undoped scaffold (BCP). For the doped scaffolds, four different doped compositions with a fixed Sr^2+^ content (6 mol%, BCP-6Sr), alone or in various combinations with other doping ions (2 mol% Mg^2+^, BCP-6Sr2Mg; 2 mol% Zn^2+^, BCP-6Sr2Zn; and 2 mol% Mg^2+^ plus 2 mol% Zn^2+^, BCP-6Sr2Mg2Zn), were prepared. These solutions were placed into a poly(tetrafluorethylene) (PTFE) stainless steel autoclave together with the CB pieces, followed by an HT at 200 °C for 24 h. Subsequently, the scaffolds obtained were washed with distilled water and dried in an oven at 40 °C. The heat treatment occurred at 700 °C using a heating rate of 0.5 °C min^−1^ for 1 h, followed by sintering at 1200 °C for 2 h at a heating rate of 2 °C min^−1^.

Posteriorly, the BCP-6Sr2Mg2Zn scaffolds were coated with two polymers: PCL (a well-known commercial polymer, CAPA™6800, Mn = 80.000 g mol^−1^) and PEU (a polymer synthesized in the laboratory, Mn = 63.000 g mol^−1^). The polymers were dissolved in dichloromethane (Sigma-Aldrich, Darmstadt, Germany) at a concentration of 5% (*w*/*v*). To improve the dissolution of PEU, the addition of 0.9% (*v*/*v*) dimethyl sulfoxide as a co-solvent was required. The impregnation of the scaffolds with the polymer solutions occurred under vacuum conditions. After removing the excess solutions by capillarity, the samples were dried in a vacuum oven to ensure total removal of the solvent.

### 2.2. Determination of the Porosity and Mechanical Properties of the Scaffolds

Porosity was determined with density measurements using the buoyancy method (Archimedes’ method). For this purpose, the scaffolds were immersed in distilled water, according to the European Standard EN 993-1. From the data related to the composition of the mineralogical phase compositions and, if necessary, the percentage of adsorbed polymer in each sample, it was possible to calculate the theoretical density of the scaffold and, consequently, its porosity [39]. Compression tests were performed using a universal testing machine (AG-IS10kN, Shimadzu, Kyoto, Japan) by applying a maximum force of 200 N perpendicular to the lamella of cubic scaffolds, with a side length of about 3 mm, at a constant crosshead speed of 0.5 mm min^−1^ under dry ambient conditions.

### 2.3. In Vitro Degradation Assay

An *in vitro* degradation profile of BCP-6Sr2Mg2Zn, BCP-6Sr2Mg2Zn-PEU and BCP-6Sr2Mg2Zn-PCL scaffolds (~10 mm × 10 mm) with an average thickness of 2 mm was evaluated in α-MEM medium without ribonucleosides and deoxyribonucleosides (GIBCO™ Invitrogen Corporation, Carlsbad, CA, USA) supplemented with 10% fetal bovine serum (Cytiva HyClone™ Fetal Bovine Serum (FBS) U.S. Origin, Fisher Scientific, Loughborough, UK), 1% Penicillin/Streptomycin and 1% Amphotericin B (Gibco) at pH = 7.4, according to the method previously used by Jeong and co-workers [41]. Briefly, the dried scaffolds were immersed in the culture medium at a 1:10 ratio of scaffold weight (g) to solution volume (mL) in a constant temperature incubator shaker (37 °C, 100 rpm). The samples were removed from the medium at predetermined time intervals, i.e., after 1, 7 and 14 days. The medium was renewed every 3 days. The samples were washed gently with deionized water to eliminate non-adherent particles, dehydrated with absolute ethanol and dried in an oven (40 °C) to constant weight. The weight loss (%) at each time point was calculated using Equation (1) [41],

(1) $$\mathrm{Weight}\;\mathrm{loss}\;\left(\%\right)=\frac{\mathrm{Wo}-\mathrm{Wt}}{\mathrm{Wo}}\times 100$$

where Wo is the initial weight before the *in vitro* degradation test, and Wt is the dry weight at a predetermined time.

### 2.4. Isolation and Culture of Human Mesenchymal Stromal Cells from Umbilical Cord Matrix (hUC-MSCs)

Human umbilical cords from healthy donors were kindly donated after birth, with parental consent, by Crioestaminal Saúde e Tecnologia, Biocant Park, Portugal. The umbilical cords were stored in sterile 50 mL tubes at room temperature between 12 and 48 h before tissue processing. The samples were cut into small pieces of approximately 5 cm. The pieces were washed with sterile phosphate-buffered saline (PBS) solution to remove the blood. The umbilical veins were also washed to remove blood and blood clots. To avoid contamination by endothelial cells, the umbilical veins and arteries were also removed. The specimens were then dried in tissue culture plates to promote adhesion of the fragment to the polystyrene surface, and, after adhesion, hUC-MSC proliferation medium [α-MEM (1x) with L-glutamine and without ribonucleosides and deoxyribonucleosides (GIBCO™ Invitrogen Corporation, Carlsbad, CA, USA) supplemented with 10% FBS (Cytiva HyClone™ Fetal Bovine Serum (FBS) U.S. Origin, Fisher Scientific, Loughborough, UK), 1% Penicillin/Streptomycin and 1% Amphotericin B (GIBCO™ Invitrogen Corporation, Carlsbad, CA, USA)] was added to the cell culture plate. Samples were cultured at 37 °C, 5% CO_2_ and 95% humidity for 10 days until hUC-MSCs migrated from the umbilical cord matrix and defined colonies formed. Finally, the fragments of the umbilical cord matrix were removed, and the cells were passaged. hUC-MSCs at passages 2–4 were used in the following experiments.

### 2.5. Cytocompatibility Assessments

The scaffolds were sterilized with UV irradiation light for 15 min and pre-wetted in culture medium for 2 h before their use in cell experiments. After sterilization, the scaffolds were transferred to the culture plate and cells were seeded dropwise at a cell density of 1.5 × 10^5^ cells/well. To promote cell adhesion, the samples were incubated for 30 min before the addition of culture medium (α-MEM with L-glutamine and without ribonucleosides and deoxyribonucleosides supplemented with 10% FBS, 1% Penicillin/Streptomycin and 1% Amphotericin B). The seeded scaffolds were cultured in an incubator at 37 °C and a humidified atmosphere (5% CO_2_ and 95% air) for a period of 1–14 days. The medium was changed every 2–3 days.

#### 2.5.1. Cell Viability and Proliferation

Cells (1.5 × 10^5^ cells/well) seeded on scaffolds were cultured for 24, 48 and 72 h. At the end of each period, the scaffolds were washed with PBS solution and then a mixture of Hoechst 33342 and propidium iodide in PBS solution was added to each well and incubated for an additional 15 min at 37 °C. The Live/Dead™ Cell Imaging Kit (Invitrogen^TM^) was used to monitor live and dead cells seeded into the well plates in the presence of scaffolds. The kit contains solutions of Hoechst 33342 and propidium iodide that stain both live and dead cells. The stained cells were then observed using an inverted fluorescence microscope. Cell proliferation and the viability of seeded scaffolds were also assessed using the MTT assay (Sigma-Aldrich, Darmstadt, Germany) or the MTS assay (CellTiter 96^®^ AQueous One Solution Cell Proliferation colorimetric assay, Promega Corporation, Madison, WI, USA), according to the manufacturers’ instructions. After each time point, an MTT solution (0.5 mg/mL in PBS) or an MTS reagent (10% *v*/*v*) was added to each well, and plates were incubated for 4 h in the dark at 37 °C, in a humidified atmosphere. For the MTT assay, after incubation, the MTT reagent was removed from the wells and an acidified isopropanol solution (0.04 N HCl in isopropanol) was added to dissolve the formazan crystals. Then, 100 µL of each well was transferred to a 96-well plate, and the absorbance was measured at 570 nm. Using the MTS assay, 100 µL of culture medium containing an MTS reagent was transferred to a 96-well plate, and absorbance was measured at 490 nm. A negative control (untreated cells), i.e., cells cultured without being exposed to the scaffolds, was performed. Cell viability was calculated as the percentage of viable cells relative to the untreated control cells, which were assumed to be 100% viable. Three replicate samples were tested for each condition.

#### 2.5.2. Cell Attachment

Cell adhesion and the morphology of cells adhering to the scaffolds were studied using scanning electron microscopy (SEM). After 7 and 14 days of culture, the scaffolds seeded with the human MSCs were carefully washed with PBS and fixed in 4% paraformaldehyde solution for 1 h at 4 °C. After fixation, the samples were washed with PBS and distilled water. Samples were then dehydrated in ascending ethanol solutions (30, 50, 70 and 90%) for 15 min each and, finally, dehydrated in absolute ethanol for 30 min. The dried cell-seeded scaffolds were sputter-coated with a gold layer before visualization using SEM. SEM images were acquired at various magnifications and at an accelerating voltage of 10 kV using a high-resolution field emission scanning electron microscope with Energy-dispersive X-ray spectroscopy (EDS) and Wavelength-dispersive X-ray spectroscopy (WDS) (STEM ZEISS, Merlin, Oberkochen, Germany).

### 2.6. Osteogenic Differentiation Assessment

For osteogenic differentiation, the cells were seeded on the scaffolds (1 × 10^5^ cell/well in 24-well plate), and, after 2 days, the culture medium was replaced by an osteogenic differentiation medium. The composition of the differentiation medium was as follows: StemPro^®^ Osteocyte/Chondrocyte Differentiation Basal Medium, StemPro^®^ Osteogenesis Supplement, 1% gentamicin solution, 10 nM dexamethasone, 10 mM β-glycerophosphate, 50 μM ascorbate-2-phosphate (GIBCO™ Invitrogen Corporation, Carlsbad, CA, USA). The medium was replaced with a fresh portion every 3 days for 14 days.

#### 2.6.1. Alizarin Red S Staining

Alizarin Red S was used to assess matrix mineralization on days 7 and 14 after cell seeding. The differentiation culture medium was removed, and the scaffolds seeded with cells were washed with PBS 1x. Then, samples were incubated in cold 4% paraformaldehyde solution for 30 min. Cells were rinsed again with PBS 1x and stained with 40 mM Alizarin Red S (Sigma-Aldrich, Darmstadt, Germany) at pH 4.2 for 10 min at 37 °C. To remove nonspecific staining (i.e., not associated with calcium mineral deposits), the samples were washed out several times with distilled water. The Alizarin Red S staining of hUC-MSCs without differentiation medium was also performed to compare with cells differentiated in differentiation medium. Stained samples were imaged with a stereomicroscope (Stemi 508 Stereo Microscope, Zeiss, Germany) for qualitative analysis and examined for orange-red-colored deposits.

#### 2.6.2. Alkaline Phosphatase Activity

Alkaline phosphatase (ALP) activity was measured using the hydrolysis of *p*-nitrophenyl phosphate (*p*NPP) to *p*-nitrophenol. hUC-MSC-seeded scaffolds were washed with PBS and incubated with 20 μL of *p*NPP 0.67 M, 960 μL reaction buffer (1 M Diethanolamine and 0.5 mM of MgCl_2_, pH 9.8, alkaline phosphatase, Diethanolamine Detection Kit, Sigma-Aldrich) and 20 μL of the test sample for 30 min at 37 °C in the dark. For enzyme activity control, 20 μL of diluted alkaline buffer solution was added. The reaction was quenched with 400 μL of 0.5 M NaOH, and the absorbance of 150 μL of the resulting solution was measured at 405 nm.

### 2.7. Statistical Analysis

Experiments were performed in triplicate with two replicates in each experiment. Quantitative data are reported as mean ± standard deviation (SD). Two-way analysis of variance (ANOVA) and Student’s *t*-test were used for the statistical study. When more than two groups were compared (multiple comparisons), two-way analysis ANOVA with Tukey comparison was used. The results were considered statistically different if the *p* value was less than 0.05. Significance is indicated in the graphs. All statistical analyses were performed using the Prism software package (PRISM 5.0; GraphPad Software, San Diego, CA, USA, 2007).

## 3. Results

Scaffolds obtained from marine skeletons have been explored over the last few years as a promising material for the development of bone tissue. In our previous works [38,39,40], BCP scaffolds were obtained using hydrothermal transformation of cuttlefish bones, and Sr^2+^, Mg^2+^ and/or Zn^2+^ ions were incorporated by partial replacement of calcium or by coating bioactive glass derived from sol-gel. The mechanical properties of the hydrothermally transformed samples were improved by applying PCL or PEU coatings. Table 1 presents the main physical-mechanical properties of the scaffolds under study in this work. More details can be found elsewhere [39].

**Figure 1:**
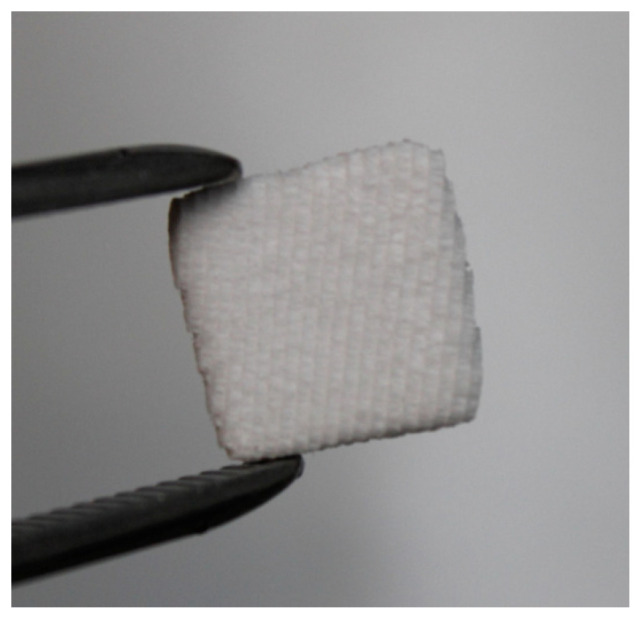
Representative image of the coated doped scaffolds (top view).

It is important to highlight that the polymer coatings did not compromise the porosity of the scaffolds. In this work, the potential use of coated doped scaffolds (Figure 1) [38,39,40] in promoting the differentiation of hUC-MSCs into an osteoblastic lineage was evaluated.

### 3.1. Human MSCs Seeded onto BCP-6Sr2Mg2Zn Scaffolds Coated with PCL and PEU Show High Cell Viability Rates

**Figure 2:**
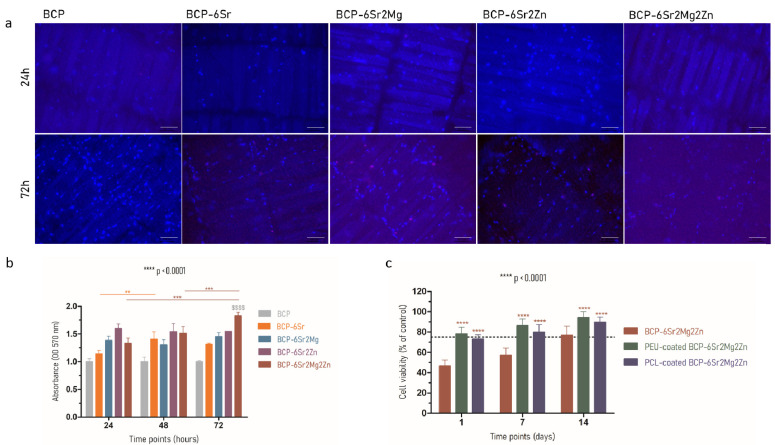
(**a**) Live/Dead fluorescence images after 24 and 72 h of cell culture. The nuclei of healthy cells were stained with Hoechst 33342 (blue), and dead cells were stained with propidium iodide (red). Scale bar represents 100 µm. (**b**) Viability results from undoped and doped scaffolds. Data represent mean ± SD, n = 3. The * indicates significant difference (*** *p* < 0.001 and ** *p* < 0.05) in the same sample at different times of culture and the ^$^ denotes significant difference between the samples on the same culture day (^$$$$^ *p* < 0.0001). (**c**) Viability results from the doped (un)coated scaffolds. Data represent mean ± SD, n = 5 (asterisks denote significant differences relative to the control group BCP-6Sr2Mg2Zn, **** *p* < 0.0001).

The first set of experiments investigated whether doping elements introduced by partial replacement of calcium would have a positive effect on the viability and proliferation of hUC-MSCs in direct contact with (un)doped scaffolds after 24, 48 and 72 h of incubation. The Live/Dead cell assay was used to directly monitor the proportion of viable and dead cells upon contact with the undoped and doped BCP scaffolds. The fluorescence microscopy images suggest that hUC-MSCs remained viable over 72 h of culture for all examined scaffolds (Figure 2a). Viable cells (stained with Hoechst 33342) were present in large numbers as detected by the high bright blue fluorescence in the experiments. Very few dead cells (stained with propidium iodide), which are red in color, were observed in the images (Figure 2a). Taken together, both tests show that the undoped and doped scaffolds are minimally cytotoxic and do not negatively affect cell viability. The results from the viability tests also indicate that the cells seeded on the BCP-6Sr2Mg2Zn scaffolds exhibited higher cell viability compared to the neat BCP scaffolds after 72 h of incubation (**** *p* < 0.0001) (Figure 2b). Furthermore, the cells’ proliferation levels on the BCP-6Sr2Mg2Zn samples were significantly greater than those obtained at 24 h (* *p* < 0.05). There were no statistically significant differences between any of the samples based on Student *t*-test results (Figure 2b). All doped scaffolds are non-toxic and cytocompatible (cell viability > 5%).

Considering that the BCP-6Sr2Mg2Zn scaffolds exhibited the highest cell viability, it was decided to proceed with additional *in vitro* cytotoxicity tests on the scaffolds coated with PCL and PEU. To this end, hUC-MSCs were seeded onto the surface of PCL-coated BCP-6Sr2Mg2Zn and PEU-coated BCP-6Sr2Mg2Zn scaffolds, and cell proliferation studies were performed at different time points over a 14-day period. For comparison purposes, the BCP-6Sr2Mg2Zn scaffold was also subjected to the same test. As shown in Figure 2c, the uncoated samples (BCP-6Sr2Mg2Zn) showed a tendency toward increased cell viability over time, but the PCL- and PEU-coated scaffolds resulted in superior cell viability at all time points, indicating that the polymer coatings did not negatively affect cell behavior over time. One-way ANOVA and Tukey’s multiple comparison tests revealed a statistically significant increase in the viability of cells grown on BCP-6Sr2Mg2Zn scaffolds coated with PCL and PEU compared to cells on BCP-6Sr2Mg2Zn scaffolds (**** *p* < 0.0001) (Figure 2c). While 73.08 ± 4.41% and 78.10 ± 6.74% of seeded cells were attached in PCL-coated BCP-6Sr2Mg2Zn and PEU-coated BCP-6Sr2Mg2Zn, respectively, after 1 day, only 46.52 ± 6.03% of cells were attached in BCP-6Sr2Mg2Zn (Figure 2c). On the seventh day of incubation, statistically significant increases were observed between PCL-coated BCP-6Sr2Mg2Zn (79.86 ± 7.42%) and PEU-coated BCP-6Sr2Mg2Zn (86.32 ± 6.56%) scaffolds compared to their uncoated counterparts (57.20 ± 7.02%) (Figure 2c). Finally, on the 14th day of incubation, there was, again, a significant difference in supporting cell growth on the scaffolds between the BCP-6Sr2Mg2Zn scaffolds (76.73 ± 9.36%) and the BCP-6Sr2Mg2Zn scaffolds coated with PCL (89.75 ± 5.01%) and PEU (94.31 ± 5.86%). Briefly, the metabolic activity of hUC-MSCs on BCP-6Sr2Mg2Zn scaffolds coated with PCL and PEU was higher than BCP-6Sr2Mg2Zn samples after 1, 7 and 14 days of culture. So, the viability results showed that the PCL and PEU coating process seems to, in fact, improve cell viability. Together, this indicates that the scaffolds are cytocompatible and able to support the viability of hUC-MSCs after their attachment.

### 3.2. hUC-MSCs Show Normal Morphology and Adhesion Properties When Growing onto BCP-6Sr2Mg2Zn Scaffolds Coated with PCL and PEU Scaffolds

**Figure 3:**
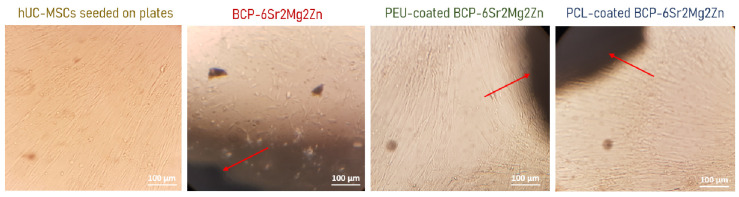
Inverted microscopic images (scale bar, 100 µm) of hUC-MSCs after 14 days of culture: control group (regular cell culture plate), BCP-6Sr2Mg2Zn, PCL-coated BCP-6Sr2Mg2Zn and PEU-coated BCP-6Sr2Mg2Zn scaffolds. The black specks in the figures are the shadows of the scaffolds, so the red arrows indicate the presence of scaffolds on the culture plates.

Cell growth was also monitored with morphological/adhesion studies using inverted and electron microscopes. The process of cell adhesion involves four stages: attachment, filopodial growth, cytoplasmic web and flattening of the cell mass. It depends on several factors such as surface topography, surface chemistry, surface energy, and hydrophilicity, as well as the chemical composition of the sample [42]. Although some morphological heterogeneity was observed in the adherent fraction during the initial culture period, morphological homogeneity was gradually achieved over time. According to the inverted microscope images (Figure 3), hUC-MSCs seeded on the surface of BCP-6Sr2Mg2Zn scaffolds coated with PCL and PEU showed a similar morphology to control cells (hUC-MSCs seeded on plates). After 14 days in culture (Figure 3), the cells were viable, exhibiting a normal, spread and fusiform morphology characteristic of this cell type (fibroblast-like), and no spontaneous differentiation was observed. It can also be seen that hUC-MSCs tend to grow toward the scaffolds (red arrows point to scaffold, i.e., the black shadow seen in the figures is the scaffold). In turn, the cell density in the BCP-6Sr2Mg2Zn scaffolds was lower, and the morphology showed an unhealthy appearance (spheroidal shape) compared to the control group (hUC-MSCs seeded on plates).

**Figure 4:**
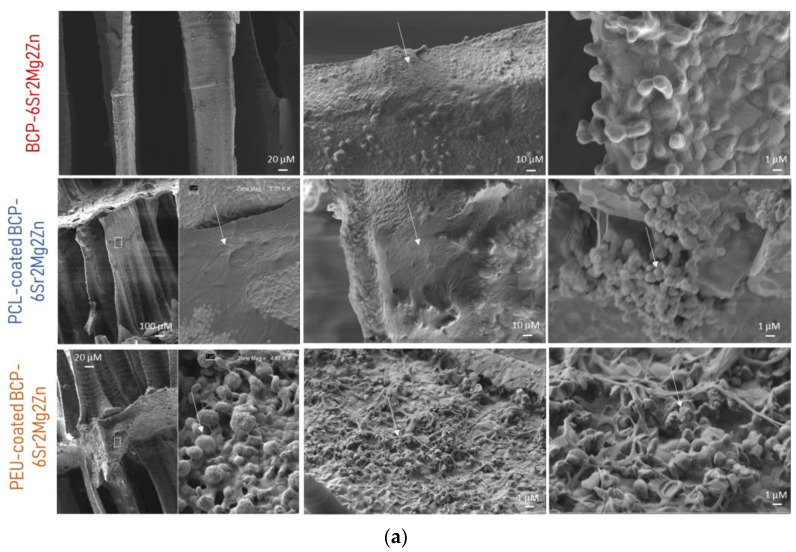

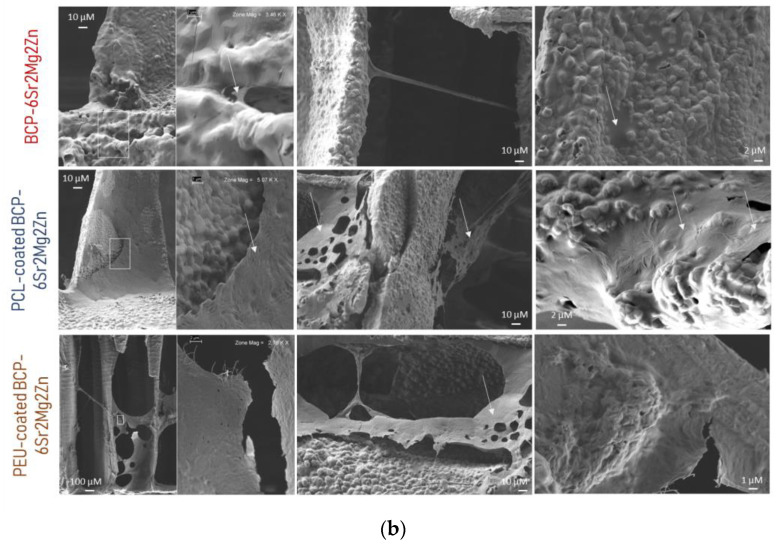
(**a**) Representative scanning electron micrographs of cell attachment to BCP-6Sr2Mg2Zn and BCP-6Sr2Mg2Zn scaffolds coated with PCL and PEU after 7 days of culture. (**b**) Representative scanning electron micrographs of cell attachment to BCP-6Sr2Mg2Zn and BCP-6Sr2Mg2Zn scaffolds coated with PCL and PEU after 14 days of culture. Attachment of hUC-MSCs to the surface of scaffolds is highlighted with white arrows.

The adhesion, spreading and morphology of hUC-MSC cells seeded on the prepared scaffolds were also studied using SEM. Figure 4a,b show SEM images of hUC-MSCs grown on coated scaffolds after 7 and 14 days of cell culture, respectively. After 7 days of culture, hUC-MSCs proliferated well, exhibited a flat appearance and formed confluent monolayers, indicating that our scaffolds supported uniform cell attachment and growth. In the high-magnification images in Figure 4a referring to the PEU-coated BCP-6Sr2Mg2Zn scaffold, it can be seen that the hUC-MSCs exhibit a partially spread morphology with some filipodial protrusions, indicating that the cells are starting to interact with the substrate. In turn, the surface of the PCL-coated BCP-6Sr2Mg2Zn scaffold is covered by a layer of cells, making it difficult to draw conclusions about the morphology of the cells. After 14 days of culture, the surface of the BCP-6Sr2Mg2Zn scaffolds coated with PCL and PEU was almost completely covered with dense layers of cells and the extracellular matrix secreted by them (Figure 4b), suggesting better spreading of the cells. The cell density on the BCP-6Sr2Mg2Zn scaffolds was lower and the formation of a cell layer is not visible. Only single cells exist on the scaffold surface.

In Figure 4b, the cells cultured for 14 days grew in colonies on the surface and showed a greater number of cells with the formation of more clusters. The surface of the coated doped scaffolds was almost completely covered with dense layers of hUC-MSCs, which formed smooth, mesh-like structures (the entire surface was covered), in contrast to the rough surface of the BCP-6Sr2Mg2Zn scaffold. hUC-MSCs showed adhesion to the scaffold itself by forming single-layered sheets. In some cases, cell sheets branched through open pores to reach the opposite edges of the pore walls and spread further over a larger part of the scaffold surface (Figure 4b). In addition, cells were observed bridging scaffold filaments, suggesting that coated scaffolds are capable of supporting cell growth and proliferation. The observed results suggest that the coated scaffolds promote intercellular contact and spatial arrangement of cells. Finally, the performed analyses suggest good cell viability, proliferation and distribution, pointing out a good cytocompatibility profile of the PEU-coated BCP-6Sr2Mg2Zn scaffold in contact with hUC-MSCs, providing a suitable environment for cell growth.

### 3.3. In Vitro Degradation

**Figure 5:**
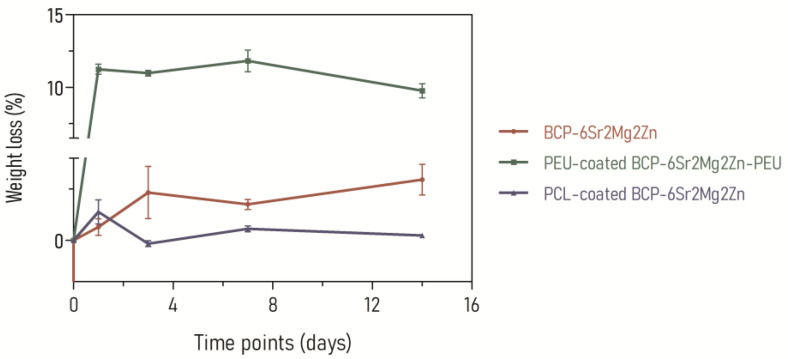
*In vitro* degradation curves of BCP-6Sr2Mg2Zn, PEU-coated BCP-6Sr2Mg2Zn and PCL-coated BCP-6Sr2Mg2Zn porous scaffolds at 1, 3, 7 and 14 days in serum-containing medium.

The *in vitro* degradability of the uncoated and coated doped scaffolds was investigated in serum-enriched culture medium (α-MEM with L-glutamine and without ribonucleosides and deoxyribonucleosides supplemented with 10% FBS, 1% Penicillin/Streptomycin and 1% Amphotericin B). Figure 5 shows the degradation curves of the BCP-6Sr2Mg2Zn, PCL-coated BCP-6Sr2Mg2Zn and PEU-coated BCP-6Sr2Mg2Zn scaffolds after 1, 3, 7 and 14 days. As shown in Figure 5, the weight loss of the PEU-coated BCP-6Sr2Mg2Zn samples was measured to be 11.25 ± 0.35% after 24 h of incubation in serum-containing medium, indicating a slightly faster degradation. On the other hand, under the same conditions, the weight loss was 0.66 ± 0.40% and 1.39 ± 0.58% for BCP-6Sr2Mg2Zn and PCL-coated BCP-6Sr2Mg2Zn scaffolds, respectively. After 3 days of incubation, the *in vitro* degradation rate of BCP-6Sr2Mg2Zn scaffolds increased, and the weight loss was 2.33 ± 1.27%. In turn, the coated scaffolds maintained their degradation percentage at 3 days of incubation: 10.98 ± 0.19% for PEU-coated BCP-6Sr2Mg2Zn scaffolds and 0.15 ± 0.14% for PCL-coated BCP-6Sr2Mg2Zn scaffolds. Compared to other scaffolds, PEU-coated BCP-6Sr2Mg2Zn scaffolds continued exhibiting a higher weight loss of 11.82 ± 0.74% after 7 days of incubation: 0.57 ± 0.14% for PCL-coated BCP-6Sr2Mg2Zn scaffolds and 1.75 ± 0.24% for BCP-6Sr2Mg2Zn scaffolds. After 14 days of incubation, the average percentage of degradation for PEU-coated BCP-6Sr2Mg2Zn scaffolds was 9.77 ± 0.48% (Figure 5). The weight loss was 2.96 ± 0.75% and 0,24 ± 0.01% for BCP-6Sr2Mg2Zn and PCL-coated BCP-6Sr2Mg2Zn scaffolds, respectively.

### 3.4. hUC-MSCs Growing onto BCP-6Sr2Mg2Zn Scaffolds Coated with PCL and PEU Scaffolds Can Differentiate into Osteoblasts

**Figure 6:**
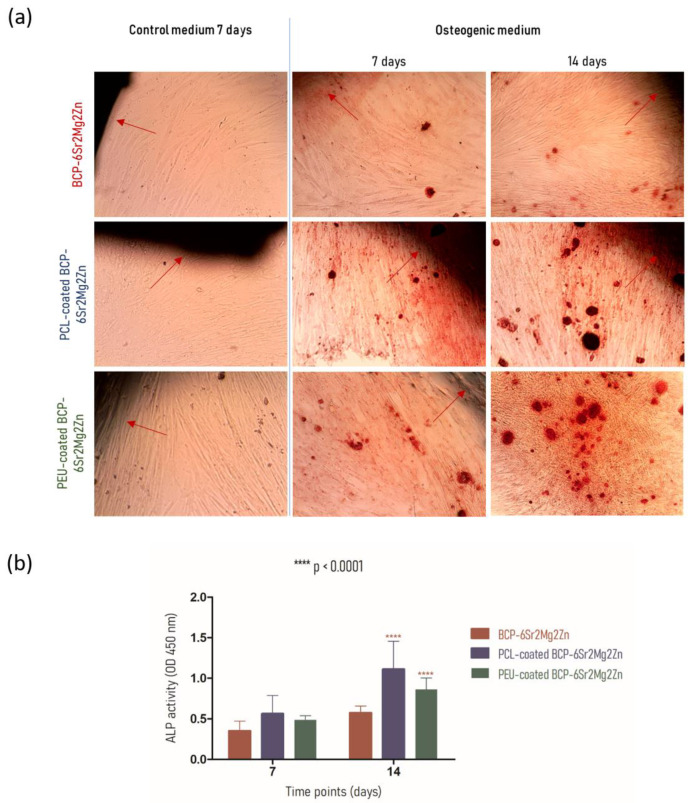
Induced differentiation of hUC-MSCs. (**a**) Osteogenic differentiation of hUC-MSCs with Alizarin Red to assess mineralization. hUC-MSCs were cultured in normal growth medium for 7 days followed by osteogenic medium during 7 and 14 days. Red arrows point to the scaffold. Dark red staining denotes mineralization. Original magnification: ×100. (**b**) Alkaline phosphatase (ALP) activity of hUC-MSCs that were cultured on the BCP-6Sr2Mg2Zn, PCL-coated BCP-6Sr2Mg2Zn and PEU-coated BCP-6Sr2Mg2Zn scaffolds after 7 and 14 days in osteogenic culture medium (asterisks denote significant difference between the groups at *p* < 0.0001).

To assess the effect of scaffold composition on the ability of hUC-MSCs to promote matrix mineralization, Alizarin Red S staining assay was used [43,44]. This staining can reveal the presence of calcium deposits, which is indicative of the differentiation of hUC-MSCs into osteoblasts (osteogenesis) [44]. When the cells reached 90% confluence (~3 days) and exhibited a fusiform shape, the basal medium was removed and osteogenic differentiation was initiated by adding osteogenic medium. Optical microscopy images of the mineralized matrix deposits on differentiating cultures of hUC-MSCs in direct contact with the scaffolds are shown in Figure 6a. Staining with Alizarin Red S showed that hUC-MSCs on scaffolds began to form calcified nodules after 7 days of cultivation in osteogenic medium. This positive result is indicated by bright red extracellular calcium deposits. In contrast, cells in the control medium (undifferentiated) did not form calcium deposits, even after 7 days (Figure 6a). After 14 days of osteogenic differentiation, cells seeded on the coated scaffolds showed a more intense Alizarin Red S staining (significantly darker) than in the BCP-6Sr2Mg2Zn scaffolds. The results indicate that the hUC-MSCs on these scaffolds produced a high number of larger calcified nodules (calcium content) that promote osteoblastic activity. hUC-MSCs in direct contact with coated scaffolds synthesized bone mineral nodules, as demonstrated with Alizarin Red staining, indicating that exposure to the coating promoted/increased the ability of hUC-MSCs to differentiate along the osteogenic lineage. The results suggest that the mineralization was mediated by the hUC-MSCs and was not the result of nonspecific calcium precipitation.

In addition to the calcium deposits stained with the Alizarin Red assay, the potential of the scaffolds for the differentiation process was confirmed by the activity of alkaline phosphatase (ALP) at 7 and 14 days (Figure 6b). ALP plays an important role in the bone mineralization process, and its activity is a phenotypic marker for osteoblastic cells [45,46]. As shown in Figure 6b, the intracellular ALP activity of hUC-MSCs grown on coated scaffolds increased with the increasing cultivation time between Days 7 and 14, indicative of the process of osteogenic differentiation. No statistically significant difference was observed between groups in the first week after induction, but, after 14 days, hUC-MSCs seeded on coated scaffolds showed significantly higher ALP activity compared with BCP-6Sr2Mg2Zn. On Day 14, ALP activity continued to increase in coated scaffold cultures and was significantly higher than in uncoated scaffold cultures (BCP-6Sr2Mg2Zn differentiated, 0.575 ± 0.082; PCL-coated BCP-6Sr2Mg2Zn differentiated, 1.113 ± 0.343; PEU-coated BCP-6Sr2Mg2Zn differentiated, 0.860 ± 0.141; **** *p* < 0.0001) (Figure 6b). The data revealed that cells in contact with PCL-coated BCP-6Sr2Mg2Zn and PEU-coated BCP-6Sr2Mg2Zn exhibited 1.9- and 1.5-fold higher ALP activity, respectively, compared to the BCP-6Sr2Mg2Zn scaffold, corroborating with the previous results of the biomineralization assays.

## 4. Discussion

In our previous work, BCP scaffolds obtained using hydrothermal transformation of cuttlefish bone were fabricated, doped with different ions (Sr^2+^, Mg^2+^ and Zn^2+^) and coated with PCL or PEU [39]. The results demonstrated that coated doped scaffolds exhibited many desirable characteristics, including high porosity, improved swelling profile and better mechanical performance than BCP scaffolds [39]. In the present study, our investigation focused on the potential use of coated doped scaffolds to promote the differentiation of hUC-MSCs into an osteoblastic lineage. To confirm cytocompatibility, the adhesion and viability of hUC-MSCs on the surface of the scaffolds were evaluated. Live/Dead staining showed that hUC-MSCs seeded on the surface of the doped scaffolds survived well after 3 days of culture. As shown in Figure 2a, most of the hUC-MSCs cultured on the scaffolds were alive (shown in blue), and only a few cells were dead (red fluorescence). It is important to emphasize that the presence of doping elements (Sr^2+^, Mg^2+^ and Zn^2+^) resulted in an increase in cellular metabolic activity (cytocompatibility). The viability results showed no cytotoxic effect of the scaffolds on the hUC-MSCs over a period of 3 days, and a time-dependent proliferation pattern was observed (Figure 2b). Many studies using biological activity assays have shown that BCP scaffolds doped with metals are not toxic to MC3T3-E1 cells [25,47], human umbilical vein endothelial cells [48], bone mesenchymal stem cells [49], rabbit-adipose-derived mesenchymal stem cells [50], human osteoblast-like MG-63 cells [51] and human mesenchymal stem cells [13]. Among the tested compositions, the BCP-6Sr2Mg2Zn scaffolds proved to be the most promising. Next, the impact of PCL or PEU coatings on the interaction of BCP-6Sr2Mg2Zn scaffolds with hUC-MSCs was also investigated through similar *in vitro* studies (Figure 2c). The uncoated samples (BCP-6Sr2Mg2Zn) showed a tendency toward increased cell viability over time, but the PCL- and PEU-coated scaffolds resulted in greater cell proliferation during the 14-day incubation (Figure 2c). These results suggest that the polymers used and the coating process itself do not affect the viability of hUC-MSCs as the viability of coated scaffolds after 14 days in culture was slightly above ~90%, comparable to cells exposed to uncoated scaffolds. The percentage of cell viability was above 80%, indicating that the scaffolds are not toxic to hUC-MSCs (according to ISO 10993-5). Similar results were reported with MG-63 cells in which the PCL-coated cuttlefish-bone-derived HAp scaffold improved cell proliferation, viability and adherence [52,53,54]. In turn, some research groups have shown the potential of PEUs as non-toxic and bioresorbable materials on MC3T3-E1 pre-osteoblast cells seeded in PEU-blended HAp scaffolds [55] and bone marrow-derived stromal cells seeded in PEU scaffolds [56]. The morphological and proliferative characteristics of the hUC-MSCs seeded on the surface of the coated doped scaffolds were similar to those observed when cultured in tissue culture polystyrene plates (Figure 3). Within the first week, hUC-MSCs showed a well-spread morphology along the coated scaffold pore walls and were able to bridge gaps between pores (Figure 4a). After 14 days of culture, and corroborating the results from the MTS assay, SEM images showed that the cells grew on the surface and in the pores of the coated scaffolds (Figure 4b). The cells on the PEU-coated scaffold appeared to be denser compared to those on the PCL-coated scaffold, resulting in partial or even complete closure of some pores. The SEM microscopy of the cell-seeded PEU-coated scaffolds also showed that most of the outer macropores of the PEU-coated scaffold were occluded by a continuous layer of cells. The cells extended around the pore wall and formed an extracellular matrix (Figure 4b). In our opinion, these data are very promising as they show that the PEU-coated scaffolds are not toxic and do not inhibit the metabolic activity or proliferation of hUC-MSCs after 14 days of culture. PEU-coated scaffolds presented a higher number of attached cells than PCL-coated scaffolds after 14 days of seeding, probably due to slight differences in the hydrophilicity of the two substrates and their high porosity (91.28 ± 0.24% vs. 89.27 ± 0.08%), which increases the surface area (Table 1) [39]. A crucial parameter to consider in tissue engineering is the degradation profile of the scaffolds, as this property influences the structural integrity, stability and mechanical performance of the scaffold but also cellular processes including cell proliferation and tissue growth (osteogenesis) [57]. Ideally, the scaffold should degrade at a rate compatible with new bone formation. The results showed that the weight loss of the PCL-coated BCP-6Sr2Mg2Zn scaffolds was almost negligible or did not change at all time points (Figure 5). These data are similar to other studies that indicate that PCL has a long degradation time, sometimes remaining *in vivo* years after implantation [58]. Teoh and co-workers (2009) prepared PCL and PCL-based composite scaffolds, and the results up to 6 months indicated a maximum mass loss of only about 1% and 7% for the PCL scaffolds and PCL-composite scaffolds in vivo, respectively [59]. To overcome this issue, PCL has been blended with natural polymers (e.g., collagen [60,61] or silk [62]) with high susceptibility to hydrolysis and/or enzymolysis in order to improve the biodegradation rate of the scaffolds. On the other hand, the PEU-coated BCP-6Sr2Mg2Zn scaffolds lost ~12% of their original weight over a 14-day period, demonstrating their suitability as temporary scaffolds during the bone healing process (Figure 3) [63]. The higher mass loss of PEU-coated BCP-6Sr2Mg2Zn scaffolds can be attributed to their high susceptibility to hydrolysis and/or enzymolysis [56,57,58] and their high porosity (91.28 ± 0.24% vs. 89.27 ± 0.08%), which enable the degradation medium to penetrate more easily into the scaffold (Table 1) [39]. The next study focused on the differentiation of hUC-MSCs seeded on the scaffolds into osteoblasts. hUC-MSCs synthesized bone mineral nodules in the coated scaffolds, as shown with Alizarin Red staining, indicating that the coating process did not affect the ability of hUC-MSCs to differentiate along the osteogenic lineage (Figure 6a). Cellular differentiation was also assessed by monitoring ALP enzyme activity. ALP activity is generally considered an early-stage marker for the osteoblastic phenotype and an important indicator of differentiation and mineralization [54]. A positive effect of PCL and PEU coating on proliferation, differentiation and extracellular matrix deposition was observed compared to the uncoated scaffold. Consistent with the results on cell proliferation, the activity of ALP in osteogenic media significantly increased in cultures on coated scaffolds, peaking at day 14 of culture (Figure 6b). Based on these results, the coated scaffolds prepared in this study show cytocompatibility and provide suitable conditions for the attachment and growth of hUC-MSCs towards a mature and differentiated state, signifying their promising potential to be candidates for regenerative medicine in the treatment of bone defects. A positive effect of PEU coating on adhesion and proliferation of hUC-MSCs was observed, i.e., hUC-MSCs adhered, proliferated well and remained viable after 14 days of culture. The PEU-coated scaffold was shown to have a continuous ‘blanket’ of cells across its surface, indicating that it is a good substrate for hUC-MSC proliferation. In short, our study suggests that PEU can be used as an alternative to PCL in bone regeneration, as it provides a suitable local microenvironment for maximal induction of osteogenesis. However, more studies are needed to realize these promises and further validate PEU’s effectiveness in bone regeneration. We plan to carry out research with the PEU-coated BCP-6Sr2Mg2Zn scaffold to repair small-sized bone defects in animal models, to fully evaluate the biological performance of these scaffolds and to validate their future clinical application. Furthermore, the PEU-coated BCP-6Sr2Mg2Zn scaffold can be further functionalized with several growth factors such as TGF-β and VEGF with sequential release.

## 5. Conclusions

In the present study, we demonstrated that the coated, doped BCP scaffolds obtained from the hydrothermal transformation of cuttlefish bones are not cytotoxic and provide adequate support for the adhesion and proliferation of hUC-MSCs. First, we demonstrated that the presence of Sr^2+^, Mg^2+^ and Zn^2+^, even at low levels, promoted better cell proliferation. The polymer-coated scaffolds led to superior cell viability rates after 7- and 14-day incubation compared to uncoated scaffolds. SEM observations showed that the hUC-MSCs seeded on the PEU-coated scaffolds proliferated well, adhered to the scaffold surfaces and spread over the scaffold surface. The PEU-coated scaffold enhanced cell differentiation ability without negatively affecting cell proliferation under *in vitro* conditions.

In short, our study suggests that PEU can be used as an alternative to PCL in bone regeneration because it provides a suitable local microenvironment for maximal induction of osteogenesis. It would be of interest to include growth factors that control cell proliferation and osteogenic differentiation in the formulation. In addition, it will be important to conduct further studies with the PEU-coated BCP-6Sr2Mg2Zn scaffold to repair small bone defects in animal models, fully evaluate the biological performance of these scaffolds and validate their future clinical application.

## Figures and Tables

**Table 1:** Physical-mechanical properties of the scaffolds under study in this work [39].

Scaffold	Porosity (%)	Mechanical Properties	
		Compressive Strength (MPa)	Young’s Modulus (MPa)
BCP	92.73 ± 0.27	0.21 ± 0.02	0.33 ± 0.03
BCP-6Sr	92.56 ± 0.35	0.20 ± 0.02	0.27 ± 0.02
BCP-6Sr2Mg	92.85 ± 0.50	0.40 ± 0.02	0.51 ± 0.09
BCP-6Sr2Zn	92.85 ± 0.54	0.20 ± 0.01	0.21 ± 0.01
BCP-6Sr2Mg2Zn	92.76 ± 0.26	0.36 ± 0.02	0.52 ± 0.05
PCL-coated BCP-6Sr2Mg2Zn	89.27 ± 0.08	0.46 ± 0.04	0.66 ± 0.05
PEU-coated BCP-6Sr2Mg2Zn	91.28 ± 0.24	1.05 ± 0.12	1.54 ± 0.18

## Data Availability

Data available on request from the corresponding author.
